# Combination therapy of chitosan, gynostemma, and motherwort alleviates the progression of experimental rat chronic renal failure by inhibiting STAT1 activation

**DOI:** 10.18632/oncotarget.24125

**Published:** 2018-01-10

**Authors:** Wenxia Bai, Shudong Wang, Shanshan An, Mengjie Guo, Guangming Gong, Wenya Liu, Shaoxin Ma, Xin Li, Jihua Fu, Wenbing Yao

**Affiliations:** ^1^ Jiangsu Key Laboratory of Druggability of Biopharmaceuticals, School of Life Science and Technology, China Pharmaceutical University, Nanjing, China; ^2^ Department of Physiology, China Pharmaceutical University, Nanjing, China; ^3^ Department of Pharmaceutics, Jinling Hospital, Nanjing University School of Medicine, Nanjing, China; ^4^ School of Medicine and Life Sciences, Nanjing University of Traditional Chinese Medicine, Nanjing, China

**Keywords:** chronic renal failure, STAT1

## Abstract

This study aimed to investigate the effect of single and combination therapy using chitosan (K), gynostemma (J), and motherwort (Y) on an experimental rat model of chronic renal failure (CRF) induced by adenine and the underlying mechanisms. CRF rats were treated with individual or combinational therapy with two or three of these agents. Biochemical indicators showed that the levels of blood urea nitrogen, creatinine and uric acid decreased and the levels of albumin and hemoglobin increased by single or combination therapy of these drugs. Drug treatment also decreased oxidative stress damage of renal tissues in CRF rats. Histopathological lesions were attenuated in each drug treatment group by various degrees. Additionally, drug treatment affected the expression of extracellular matrix (ECM) proteins including plasminogen activator inhibitor 1, collagen I, matrix metalloprotease-1, and tissue inhibitor of metalloproteinases 1. In particular, the combination therapy of K, J, and Y was superior to the respective monotherapy, which supported the prescription of KJY combination. We further studied the inhibitory effect of KJY on LPS-induced inflammation in RAW264.7 macrophages. The results showed that KJY inhibited LPS-induced secretion of inflammatory cytokines (Interferon-gamma, Interleukin-1 Beta, chemokine (C-X-C motif) ligand 10, cyclooxygenase-2 and Tumor necrosis factor-α in RAW264.7 macrophages. Combination therapy of KJY suppressed the protein expression of Cyclooxygenase-2 and inducible nitric oxide synthase *in vivo* and *in vitro*. Further study indicated that KJY inhibited STAT1 activation by down regulating p-STAT1 to exert anti-inflammatory effect and improve renal function in rats with chronic renal failure.

## INTRODUCTION

Chronic renal failure (CRF) is characterized by decreased glomerular filtration rate and other renal dysfunctions leading to metabolic disorders and clinical symptoms [1]. High serum level of blood urea nitrogen (BUN) and creatinine (CRE) are hallmarks of renal function abnormalities in CRF [2]. A diverse range of herbs have been used in single or multiple prescriptions to treat patients with chronic kidney diseases (CKD) [3], including CRF [4]. Because many diseases have intricate pathogenic factors, traditional Chinese medicine (TCM) using combinational therapeutic strategies offers great advantages [5]. Mutual enhancement of drugs is one of the principles of using herbal drugs in combination in TCM; it is achieved by using several herbs with similar or complement curative properties. In a prescription of multiple drugs to achieve mutual enhancement, there is one principal agent that exerts the main therapeutic action, and other agents that enhance or assist the effects of that principal one [6]. Currently, the treatment of CKD using TCM is often achieved by combining TCM and western pharmacologic agents.

Leonurine is an active ingredient of the Chinese herb motherwort that promotes blood circulation, removes blood stasis, and exerts antioxidant and anti-inflammatory effects [7]. It has been reported that leonurine can lower the serum levels of BUN and CRE and protect the intracellular antioxidant machinery [7]. Gypenosides are the main chemical and pharmacological substances of the Chinese herb gynostemma. Previous clinical trials have revealed that gypenosides can significantly attenuate the levels of BUN and CRE and reduces oxidative stress [8]. Chitosan, a new type of biomedical raw material, has attracted much attention in China and abroad. It has been documented that chitosan decreases the levels of BUN and CRE and elevates the activities/levels of superoxide dismutase (SOD) and glutathione peroxidase (GSH-PX) in an adenine-induced rat model of CRF [9]. Moreover, chitosan exerts potent anti-fibrosis activity by inhibiting tissue inhibitor of metalloproteinase-1 (TIMP-1) [10].

Clinical studies have indicated that chronic renal failure is not only a series of complex biochemical reactions but also a systemic chronic inflammatory response [11]. It has been shown that proinflammatory cytokines Interleukin-1 Beta (IL-1β) and tumor necrosis factor-α (TNF-α) contribute to the progression of nephritis in animal models and patients [12, 13]. Therefore, proinflammatory cytokines play an important role in the development of renal inflammatory diseases and glomerular sclerosis. In addition, inducible nitric oxide synthase (iNOS) is closely related to inflammation and a variety of inflammatory factors can up regulate its expression [14]. Several data have shown that activation of cyclooxygenase-2 (COX-2) might play a crucial role in the pathogenesis and progression of nephropathies [15].

The signal transducers and activators of transcription (STATs) are a family of cytoplasmic proteins that involved in cellular responses induced by cytokines and growth factors, acting as signal messengers and transcription factors [16]. STAT proteins play critical roles in mediating a broad range of biological processes such as cell proliferation, survival, apoptosis, and differentiation via modulating target gene expression [17]. Among the 7 members of the mammalian STAT family, STAT1 is the earliest member discovered and it is associated with immune and inflammatory diseases [18]. A previous study confirmed that phosphorylated STAT1 is detected in inflammatory glomerulus, indicating that STAT1 signaling pathways may play a role in the pathogenesis of renal inflammation [19].

Currently, there is no report on whether chitosan, gynostemma and/or motherwort work together could achieve the coordinated treatment of CRF. In the present study, we investigate the effect of chitosan, gynostemma and motherwort using as mono-therapy or combinational therapy in the treatment of CRF in an adenine-induced CRF rat model. Furthermore, we explore the mechanisms of actions of these herbs.

## RESULTS

### KJY reduced the levels of BUN and CRE in adenine-induced rat model of chronic renal failure

**Figure 1:**
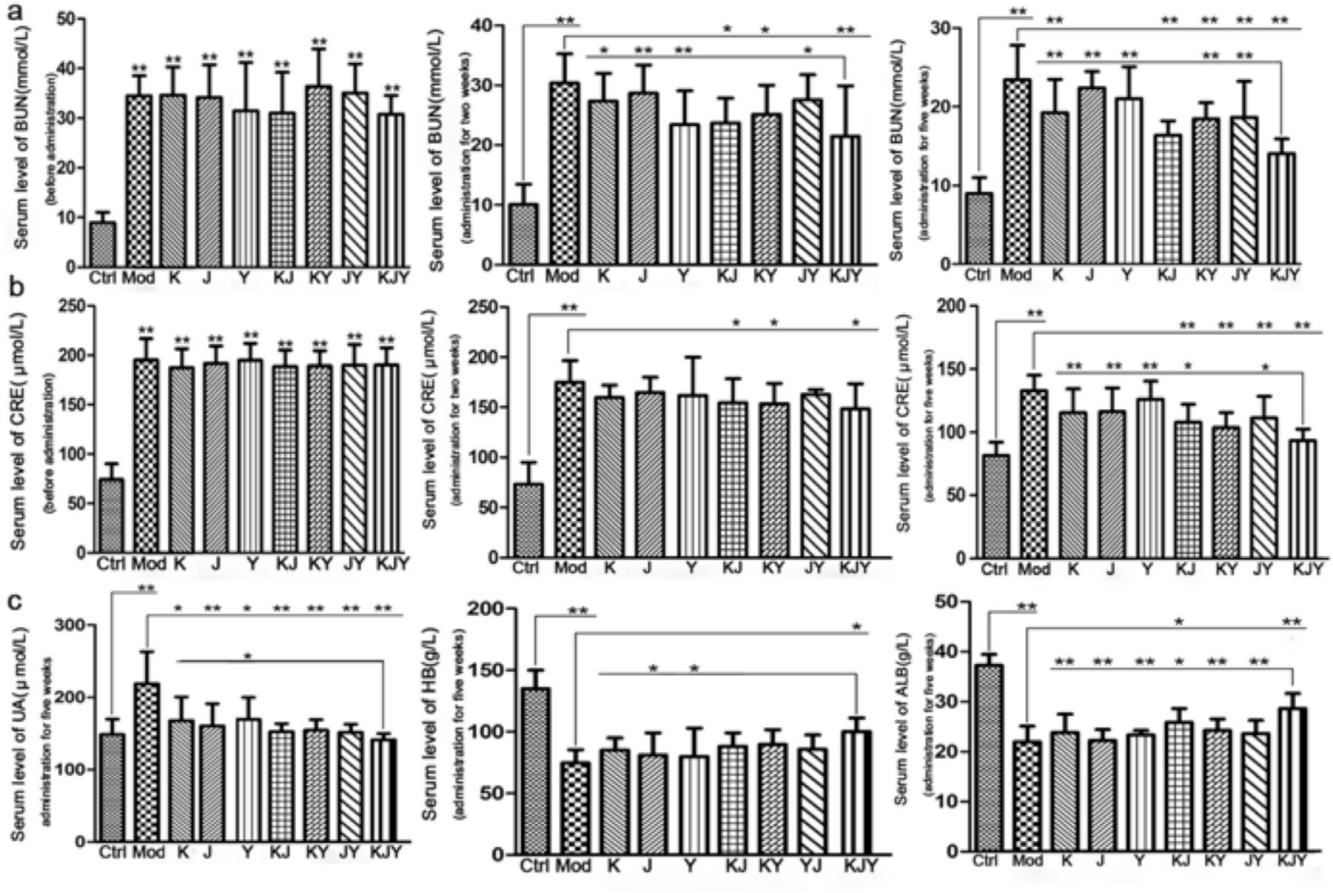
Effects of drug treatment on the serum levels of biochemical parameters in the rat model of chronic renal failure **(a)** Serum levels of BUN (left, before administration; middle, treatment for 2 weeks; right, treatment for 5 weeks). **(b)** Serum levels of CRE (left, before administration; middle, treatment for 2 weeks; right, treatment for 5 weeks). **(c)** Other biochemical parameters after treatment for 5 weeks (left, serum levels of uric acid (UA); middle, serum levels of hemoglobin (HB); right, serum levels of albumin (ALB). ^*^P<0.05, ^**^P<0.01.

CRF rat model was created. As showed in Figure 1, the levels of BUN and CRE were significantly higher in the CRF model group compared with the Ctrl group (*P<*0.01). At day 14 of drug administration, there was an obvious decrease in BUN and CRE levels in the KJ, KY, YJ and KJY treatment groups (*P<*0.05 or *P<*0.01 vs. the Mod group). After 5 weeks of continuous treatment, the levels of BUN and CRE further decreased. This result showed that these drugs can improve kidney function indicating by the increasing of BUN levels in K, KJ, KY, YJ and KJY groups. As for CRE levels, there was a strong difference between the Mod group and KJ, KY, YJ and KJY groups (*P<*0.01). Moreover, BUN and CRE levels were dramatically decreased in the KJY group (*P*<0.05 or *P*<0.01) compared to most of the other administration groups. These data suggested that an equivalent dose of KJY achieved better effects than the individual components and combination therapy using 2 of the drugs. Any 2 of the K, J, and Y achieved better efficacy than the corresponding individual treatments.

Additionally, 2 × 2 × 2 (K × J× Y) mixed-factor in ANOVA analysis revealed that K, J, and Y treatment improved the levels of BUN and CRE (*P<*0.05 or *P<*0.01). However, there was no significant interaction between K × J, K × Y, J × Y, and K × J × Y (*P*>0.05).

### KJY decreased serum levels of UA, HB, and ALB in adenine-induced rat model of chronic renal failure

The data illustrated in Figure 1 show that the levels of UA were significantly increased in experimental CRF rats (*P<*0.01 vs. Ctrl group). Administration of the drugs significantly reversed the increased UA levels (*P<*0.05 or *P<*0.01). We observed that the changes in the KJY group were more significant than those in the Y group (*P<*0.05). After CRF model was established, the levels of HB and ALB sharply decline (*P<*0.01 vs. Ctrl group). There were significant increase in ALB levels in KJY and KJ treatment groups compared to the Mod group. More importantly, increase of ALB levels in the KJY group was the greatest among all the administration groups. In addition, treatment with KJY markedly elevated HB levels.

Analysis of the 2 × 2 × 2 (K × J× Y) mixed-factor ANOVA revealed that the recovery in UA levels could be attributed to K, J, and Y (*P<*0.01). In addition, there was a significant interaction between K × J and K × Y (*P<*0.01, F=9.553; *P<*0.05, F=6.472, respectively), and particularly between K × J × Y (*P<*0.01, F=7.459). Meanwhile, there was no obvious interaction between J × Y (*P*>0.05). Regarding the effects on HB levels, K and J both achieved potent effects (*P<*0.01, *P<*0.05), whereas Y reached marginal significance (*P*<0.1). There was no significant interaction among K × J, K × Y, J × Y, and K × J × Y (*P*>0.05). The improvements in ALB levels depended on K, J, and Y. Only K × J had a significant interaction (*P<*0.05, F=4.806); but this interaction was antagonistic.

### KJY alleviated the oxidative stress damage in adenine-induced rat model of chronic renal failure

**Figure 2:**
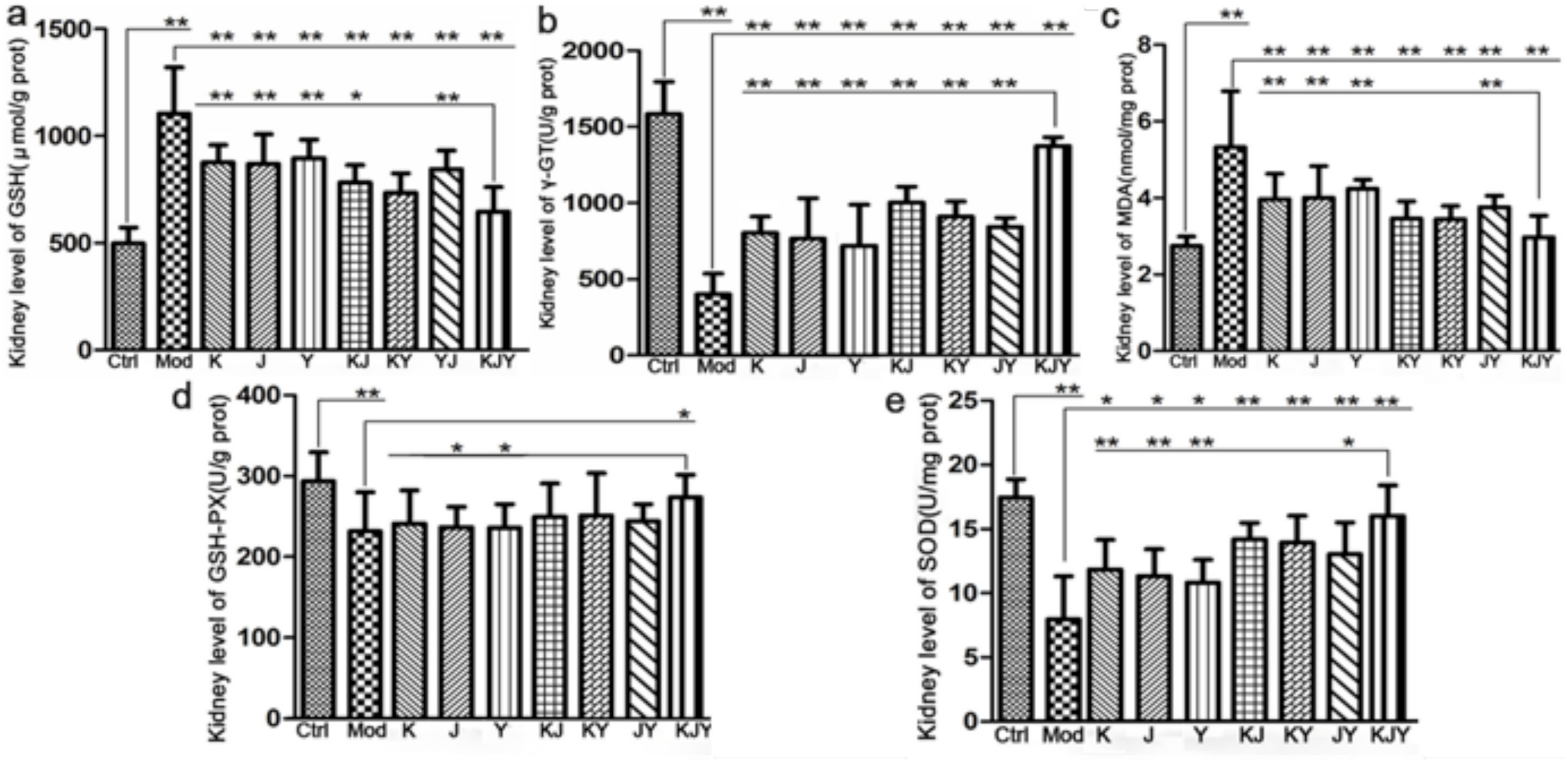
Efficacy of K, J, and Y single or combination treatments on the levels of biochemical parameters in the rat model of chronic renal failure **(a)** Concentration of malondialdehyde (MDA). **(b)** Concentration of γ-glutamyl transferase (γ-GT). **(c)** Concentration of glutathione (GSH). **(d)** Concentration of glutathione peroxidase (GSH-PX). **(e)** Concentration of superoxide dismutase (SOD). The kidney levels of biochemical parameters were measured after the administration of drugs for 5 weeks. ^*^P<0.05, ^**^P<0.01.

The results presented in Figure 2 showed that the levels of γ-GT, GSH-PX, and SOD decreased in the Mod group. However, the levels of MDA and GSH showed the contrary. During drug treatment for 5 weeks, all the drugs profoundly reversed the changes in levels of γ-GT, MDA, GSH and SOD caused by the establishment of CRF, except that GSH-PX was only significantly improved by KJY. The administration of KJY achieved the best protection against CRF as follows: it was much more effective at decreasing GSH (*P<*0.05 vs. K, J, Y, KJ, and JY) and MDA (*P<*0.05 vs. K, J, Y, and JY), as well as increasing γ-GT (*P<*0.05 vs. K, J, Y, KJ, KY, and JY), GSH-PX (*P<*0.05 vs. Y and J), and SOD (*P<*0.05 vs. K, J, Y, and JY). It was found that the combination of K, J, and Y achieved better efficacy than the individual drug treatments.

The results of 2 × 2 × 2 (K × J × Y) mixed-factor ANOVA analysis found that K, J, and Y exerted dramatic favorable changes of the aforementioned biochemical indicators, except for GSH-PX. Only K had a slight effect on GSH-PX levels (*P*<0.1). However, there was no significant interaction between K × J, K × Y, J × Y, and K × J× Y (*P*>0.05) on the index of SOD and GSH-PX. The following results revealed the combination therapy of K, J, and Y in terms of oxidative stress: J × Y exerted obvious synergistic effects on decreasing MDA and GSH levels; K × J × Y, exerted interactions to modify GSH and γ-GT levels; K × J caused a synergistic increase in γ-GT levels.

### KJY protected against renal lesions and improved renal damage of adenine-induced rat model of chronic renal failure

**Figure 3:**
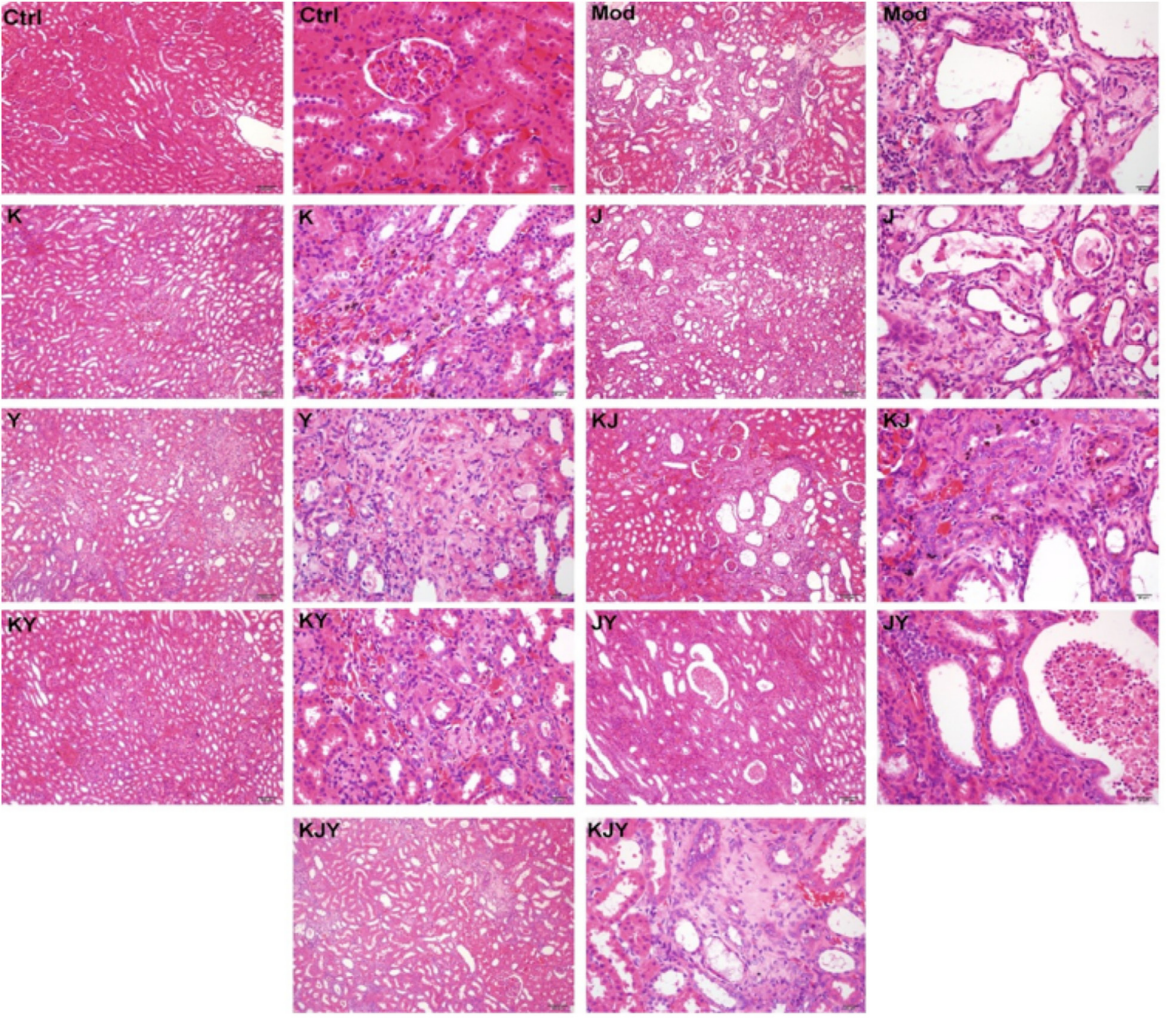
Representative histopathological micrographs of rat model of chronic renal failure with or without drug treatments for 5 weeks H&E staining. No pathologicallesions were observed in the Ctrl group. Tubular dilatation, atrophy, necrosis, chronic inflammatory cell infiltration and the marked expansion of the interstitial area and crystal deposition were observed in the Mod group. The degree of the lesions was reduced in each administration group in the following order (decreasing from heaviest to lightest): Mod > J > Y > JY > K > KJY > KJ > KY > Ctrl. Left, 100x; right, 400x;

Histopathological features of the kidney parenchyma are illustrated in Figure 3. Normal features were observed in Ctrl group, which had no lesions. After creating the CRF model, there was evidence of tubular dilatation, atrophy, and necrosis with chronic inflammatory cell infiltration in the renal interstitium. Renal tubular cells were swollen, with necrotic nuclei. There was a marked expansion of the interstitial area, together with crystal deposition. All these abnormalities revealed that the experimental CRF model was successfully established by the administration of adenine. Compared with the Mod group, the degree of the lesions in each drug administration group was as follows (decreasing from heaviest to lightest): Mod group > J group > Y group > JY group > K group > KJY group > KJ group > KY group > Ctrl group.

### KJY regulated the expression of extracellular matrix (ECM) proteins in kidney tissues of adenine-induced rat model of chronic renal failure

**Figure 4:**
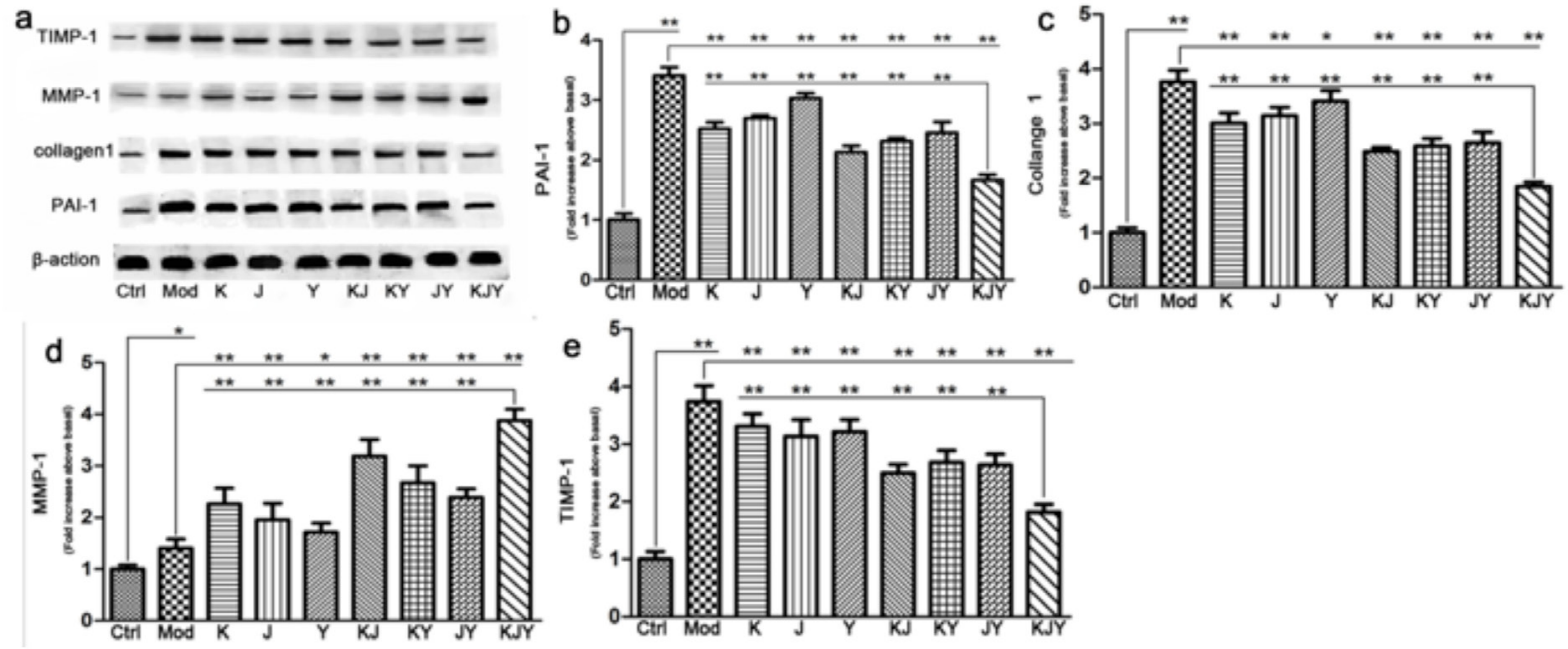
The effects of drug treatment on biomarkers of extracellular matrix in the rat model of chronic renal failure **(a)** Western blots. **(b)** PAI-1 expression. **(c)** Collagen I expression. **(d)** MMP-1 expression. **(e)** TIMP-1 expression. The intensity of the ECM mediators was normalized to β-actin. The expression of protein markers in Ctrl was set to 1, and used to normalize the expression of other markers in treatment groups. ^*^P<0.05, ^**^P<0.01.

The expression of PAI-1, collagen I, TIMP-1, and MMP-1 was increased in the established CRF model (*P<*0.05 or *P<*0.01). After 5 weeks of continuous drug treatment, the protein levels of PAI-1, collagen I, and TIMP-1 decreased in all the drug treatment groups (*P<*0.05 or *P<*0.01), whereas MMP-1 levels increased. More importantly, there were marked distinctions between the KJY group and other groups (*P<*0.01). As illustrated in Figure 4, any two combinations of K, J, or Y achieved better efficacy than each individual treatment. In general, these data confirmed that equivalent doses of KJY had a greater effect on altering the expression of these ECM proteins.

The results of 2 × 2 × 2 (K × J× Y) mixed-factor ANOVA analysis revealed that K, J, and Y influenced the expression of PAI-1, collagen I, MMP-1, and TIMP-1 (*P<*0.01). In particular, K × J and K × J × Y had a significant interaction on expression of MMP-1 (*P<*0.05, F=5.937) and PAI-1 (*P<*0.05, F=6.145), respectively. No interaction was observed for the other combinations on the expression of those 4 proteins (*P*>0.05).

### KJY inhibited the protein expression of COX-2, iNOS and p-STAT1 in renal tissues of adenine-induced rat model of chronic renal failure

**Figure 5:**
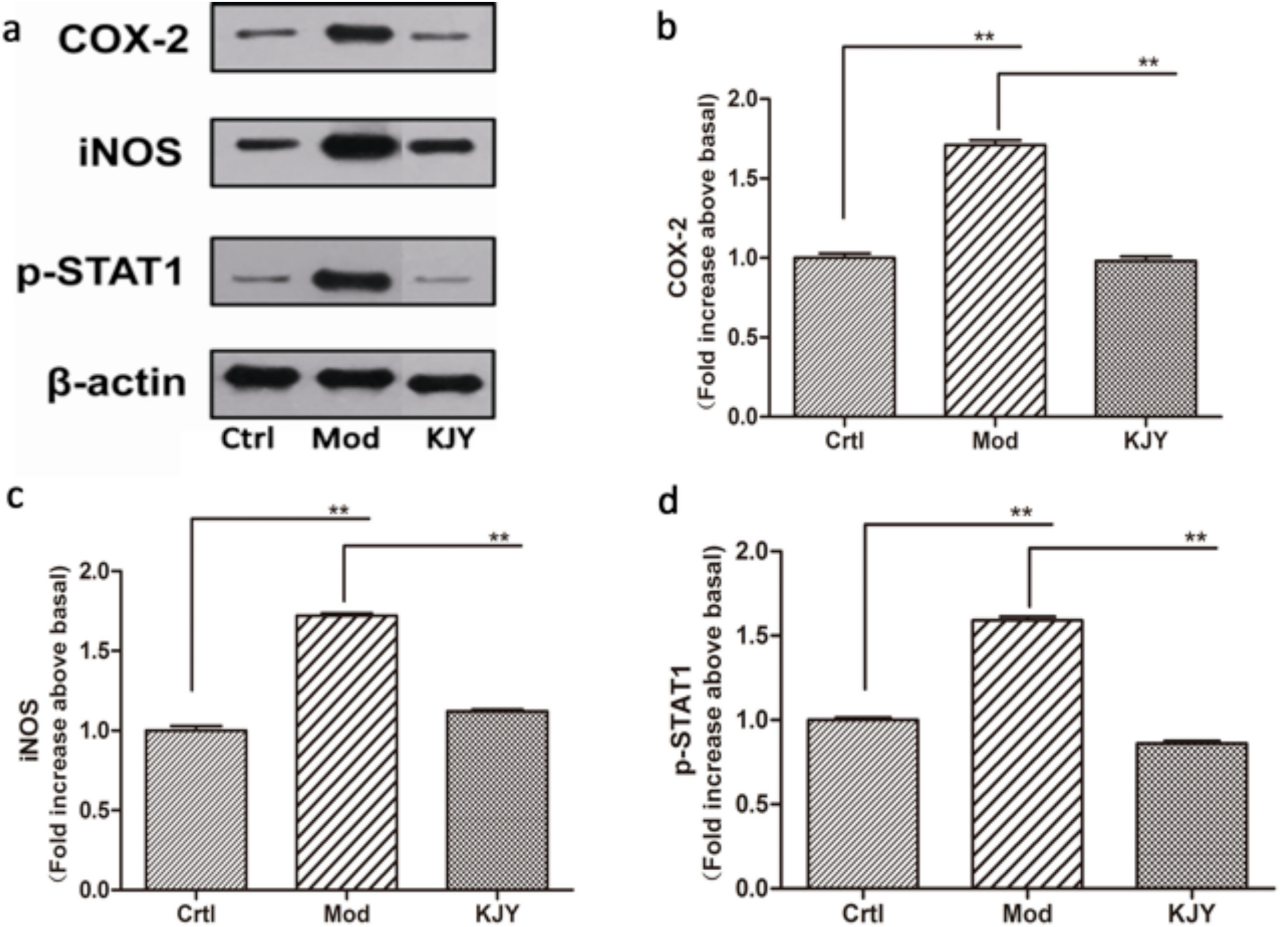
Effect of KJY on expression of COX-2, iNOS and p-STAT1 in renal tissue in the rat model of chronic renal failure **(a)** Western blots. **(b)** Cyclooxygenase-2 (COX-2) expression. **(c)** inducible nitric oxide synthase (iNOS) expression. **(d)** Phosphorylated Signal Transducer and Activator of Transcription 1 (p-STAT1) expression. The intensity of the proteins was normalized to β-actin. The expression of protein markers in Ctrl was set to 1, and used to normalize the expression of other markers in treatment groups. ^*^P<0.05, ^**^P<0.01.

The protein levels of COX-2, iNOS and p-STAT1 in the kidney tissues of rats with chronic renal failure induced by adenine were examined by Western Blotting. As shown in Figure 5, we observed that protein levels of COX-2, iNOS and p-STAT1 was down regulated after modeling, but restored after treatment with KJY (*P<*0.01).

### KJY decreased the production of IFN-γ, IL-β, CXCL10, COX-2 and TNF-α in LPS-induced RAW264.7 macrophages

**Figure 6:**
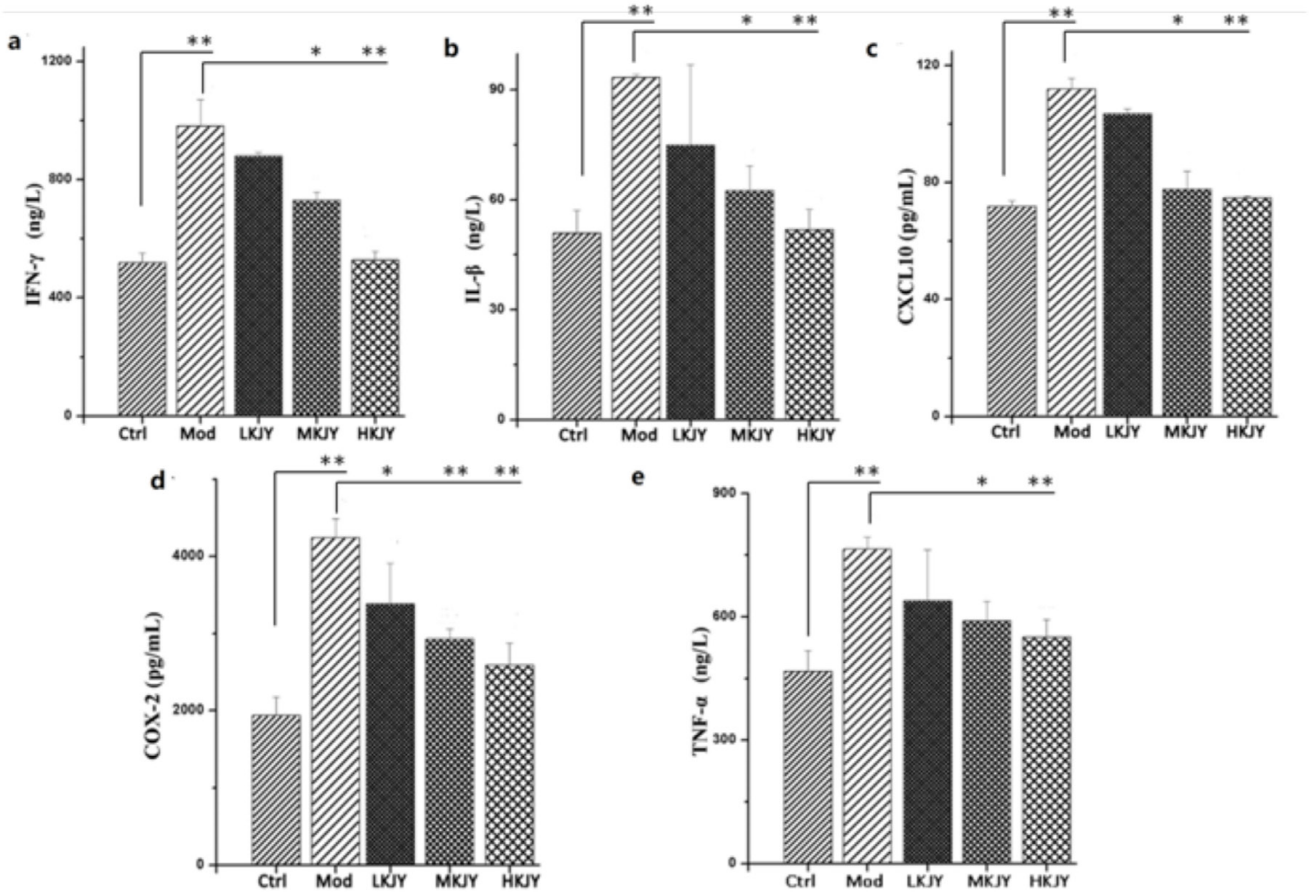
Effect of KJY on inhibiting secretion of cytokines in LPS-induced RAW264. 7 Macrophage **(a)** secretion of interferon-gamma (IFN-γ). **(b)** secretion of Interleukin-1 Beta (IL-1β). **(c)** secretion of chemokine (C-X-C motif) ligand 10 (CXCL10). **(d)** secretion of Cyclooxygenase-2 (COX-2). **(e)** secretion of Tumor necrosis factor-α (TNF-α). ^*^P<0.05, ^**^P<0.01.

MTT assay was used to investigate the effect of KJY on the cell viability of RAW264.7 macrophages. The results showed that 31∼248 μg/mL of KJY had no obvious toxic effect on LPS-induced RAW264.7 macrophages. Therefore, we choose 31, 62 and 93 μg / mL as the low, medium and high concentrations of KJY (marked as LKJY, MKJY and HKJY) for the treatment of RAW264.7 macrophages (shown in Table 1). We found that the secretion of IFN-γ, IL-β, CXCL10, COX-2 and TNF-α was significantly increased in RAW264.7 macrophages stimulated by LPS. After the treatment with KJY, the secretion of IFN-γ, IL-β, CXCL10, COX-2 and TNF-α were dramatically decreased Figure 6 (*P<*0.05).

**Table 1:** Effects of KJY on cell viability in RAW264.7 macrophages

Group	Dose (μg/mL)	Cell viability (%)
Ctrl	-	100
Mod	1	90
	31	107
	62	97
KJY	93	106
	186	102
	248	95

### KJY inhibited the transcription of COX-2 and iNOS in LPS-induced RAW264.7 macrophages

**Figure 7:**
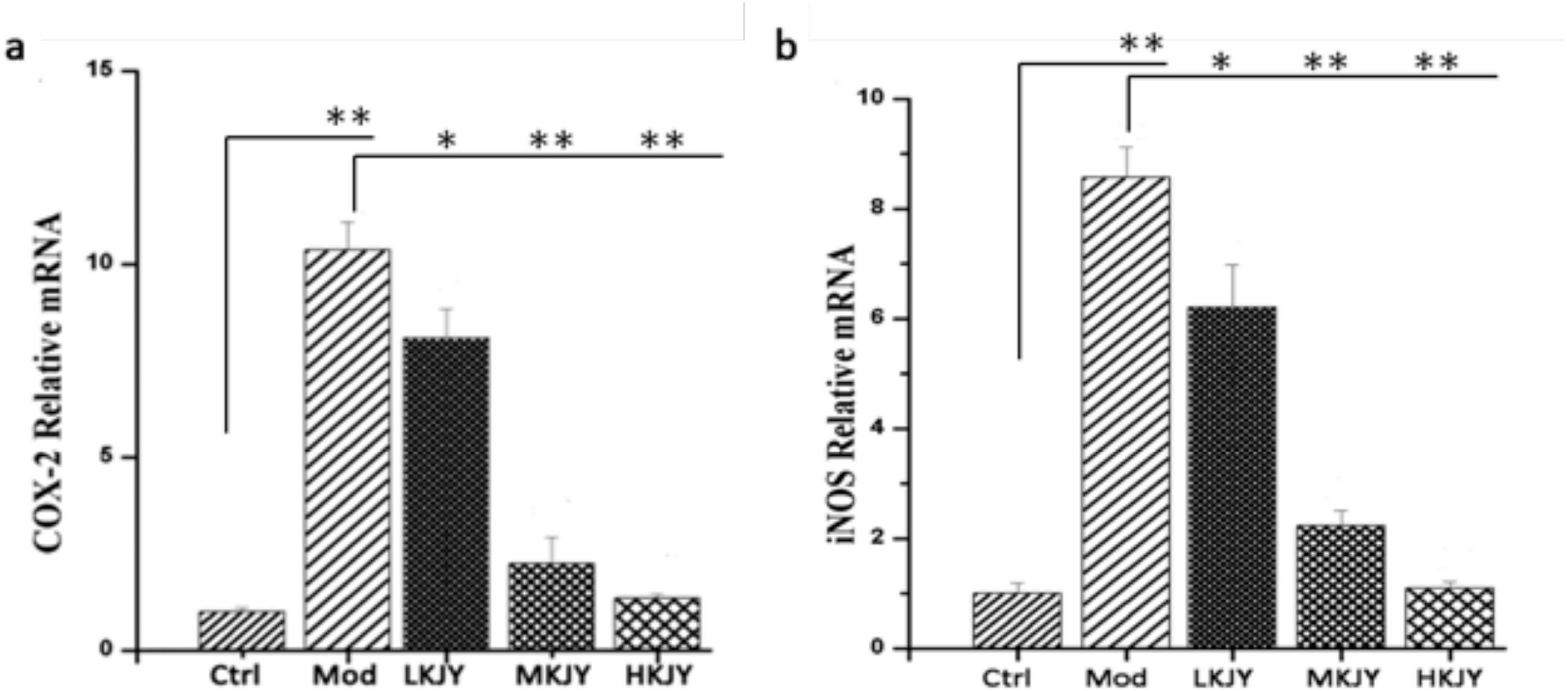
Effect of KJY on inhibiting mRNA expression of COX-2 and iNOS in LPS-induced RAW264. 7 Macrophage **(a)** mRNA level of Cyclooxygenase-2 (COX-2). **(b)** mRNA level of inducible nitric oxide synthase (iNOS).^*^P<0.05, ^**^P<0.01.

The mRNA levels of COX-2 and iNOS were up-regulated in LPS-induced RAW264.7 macrophages. Different doses of KJY significantly inhibited the transcription of COX-2 and iNOS in RAW264.7 macrophages Figure 7 (*P<*0.05).

### KJY inhibited the protein expression of COX-2, iNOS and p-STAT1 in LPS-induced RAW264.7 macrophages

**Figure 8:**
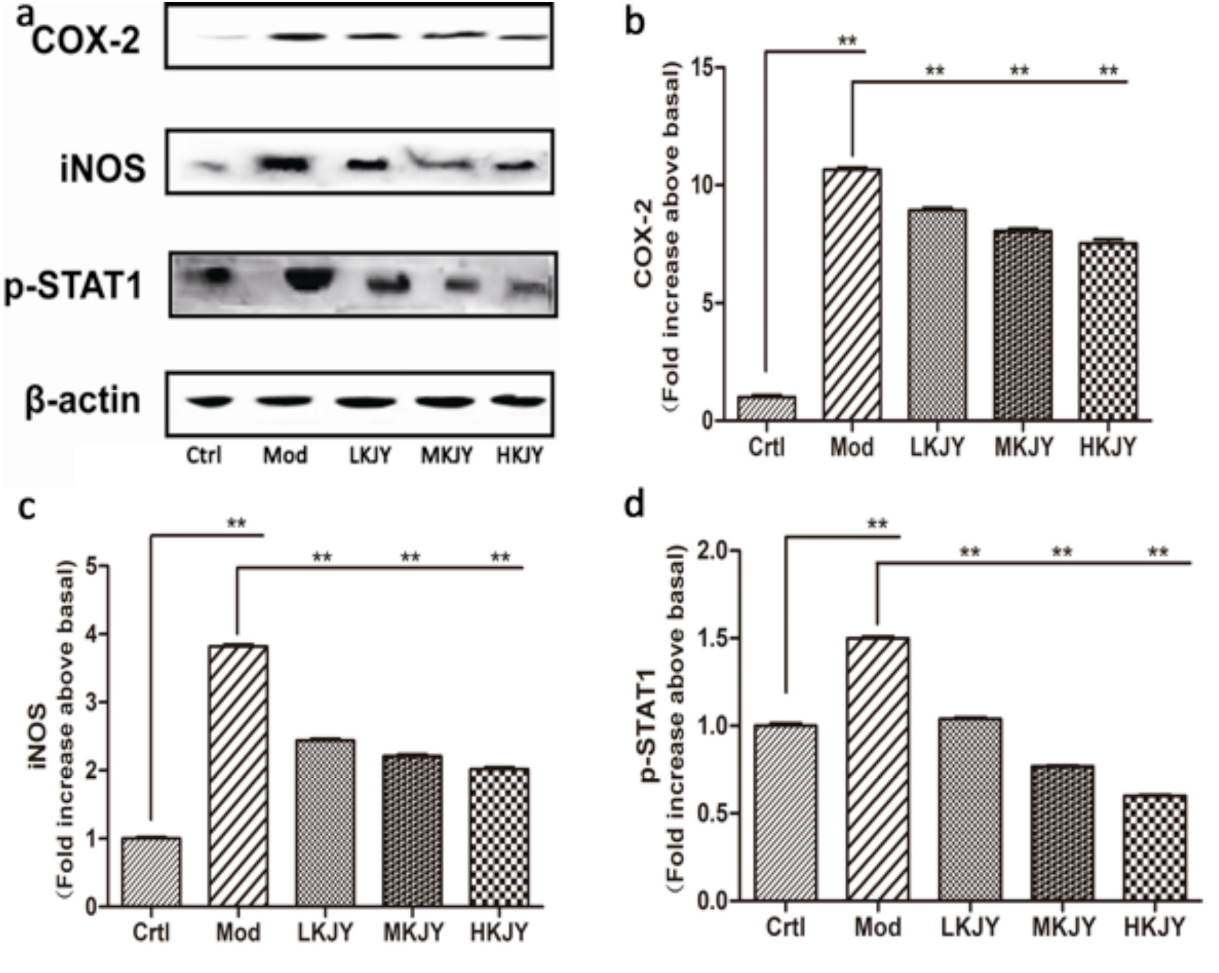
Effect of KJY on inhibiting protein expression of COX-2 and iNOS in LPS-induced RAW264. 7 Macrophage **(a)** Western blots. **(b)** Cyclooxygenase-2 (COX-2) expression. **(c)** inducible nitric oxide synthase (iNOS) expression. **(d)** Phosphorylated Signal Transducer and Activator of Transcription 1 (p-STAT1) expression. The intensity of the proteins was normalized to β-actin. The expression of protein markers in Ctrl was set to 1, and used to normalize the expression of other markers in treatment groups. ^*^P<0.05, ^**^P<0.01.

The protein levels of COX-2, iNOS and p-STAT1 were remarkably increased in LPS-induced RAW264.7 macrophages. KJY inhibited the protein expression of COX-2, iNOS and p-STAT1 in LPS-induced RAW264.7 macrophages (*P<*0.05) Figure 8.

## DISCUSSION

Chronic renal failure (CRF) is a common clinical syndrome resulted from continued diminishing renal function caused by various chronic kidney diseases (CKD) [9]. A substantial body of evidence has indicated that several Chinese herbs that possess diuretic and renal protective actions have been used in treating renal diseases to slow down the progress of CKD [21]. The mechanism of actions of these herbs are related to anti-inflammatory, anti-oxidative, and anti-fibrotic effects, as well as improved metabolic disturbances [22]. The current study was performed to investigate the efficacy of single and combinational regimen using chitosan (K), gynostemma (J), and motherwort (Y) on rats with CRF, as well as their mechanisms of actions.

As curial indicators of CKD, serum levels of BUN, CRE and UA are significantly increased in subjects with CRF [23]. These indicators in rats with CRF decreased after treatment with individual or combined drugs. This indicates that BUN, CRE, and UA were scavenged from the bloodstream, suggesting that glomerular filtration function was repaired. HB and ALB, indicators of the nutritional condition, are closely related to disease prognosis [24]. The reduction of serum ALB and HB concentration was reversed by treatment with KJY, suggesting that KJY ameliorated kidney filtration function. Shutting down oxidative stress is an important therapeutic intervention to postpone the progress of CRF. Reports have shown that SOD, MDA, GSH-PX, GSH, and γ-GT are responsible for oxidative stress [25]. The current findings suggested the significant induction of MDA and GSH and the depletion of γ-GT and SOD caused by CRF were markedly reversed by the administered drugs. However, the inhibition of GSH-PX was mitigated only by KJY. We also found that equivalent doses of KJY treatment achieved the best efficacy, and combinations of 2 drugs generally obtained greater effects than any individual drug.

It was known that insults to renal cells and membranes are caused by free radicals, leading to tubular dysfunction and interstitial fibrosis [9, 26]; this is consistent with the current pathological observations. The administration of the drugs alleviated the renal lesions by varying degrees in this study. The main characteristic of tubulointerstitial fibrosis is extracellular matrix (ECM) components, such as collagen I, III, and IV accumulation [27]. The current study focused on detecting the expression of collagen I [28]. Collagen I expression was inhibited by all the drug treatments, suggesting that the glomerular injury due to fibrosclerotic lesions was reversed [29]. Two major systems the plasminogen (PAs) system and matrix metalloproteinases (MMPs) are rich sources of ECM-degrading proteases to maintain the homeostasis of the ECM. MMP-1, which is the first enzyme in the MMP family, degrades fibril collagen [30]. TIMP-1 is a crucial tissue inhibitor of MMPs by suppressing active MMPs and preventing matrix digestion [30]. PAI-1 acts as a potent regulator of fibrinolysis by strongly inhibiting PAs [31]. In the current study, the expression of MMP-1 was largely up-regulated, along with the depletion of TIMP-1 and PAI-1 in all the administration groups. In summary, the combination of 2 or 3 drugs had a greater effect on regulating the expression of collagen I, MMP-1, PAI-1 and TIMP-1 than single treatments alone; the combination of 3 drugs KJY achieved the best effects.

When stimulated by endotoxins, such as lipopolysaccharides (LPS), various proinflammatory mediators and cytokines such as tumor necrosis factor-α (TNF-α), and interleukin (IL)-1β are released from macrophages [32]. Interferon-γ (IFN-γ), secreted mainly by activated T helper-1 (Th1) lymphocytes, plays a crucial role in the development of pro-inflammatory macrophages [33]. CXCL10, a member of the α (C-X-C) subfamily [34], has been reported to contribute to the severity of kidney diseases in several animal models of nephrosis [35]. Cyclooxygenase-2 (COX-2) is a predominant enzyme that contributes to glomerular injury [36]. Throughout the inflammatory processes, the protein expression of inducible NO synthase (iNOS) and COX-2 are notably elevated, which expedites the generation of NO and PGE2, respectively [37]. At the cellular level, these cytokines are thought to increase vascular endothelial cell permeability, contribute to glomerular hypercellularity and GBM thickening, and can be directly toxic to renal cells [38]. Our study found that the secretion of TNF-α, IL-1β, IFN-γ, CXCL10 and COX-2 was increased in LPS-induced RAW 264.7 macrophages. KJY exerted a significant inhibitory effect on production of these five cytokines in a dose-dependent manner. Moreover, both mRNA and protein expression of iNOS and COX-2 were up-regulated in LPS-induced RAW 264.7 macrophages, but blocked by KJY treatment in a concentration-dependent manner. Consistent with this, an increased expression of iNOS and COX-2 was inhibited by KJY in kidney tissue. Thus, KJY exerted anti-inflammatory effect by inhibiting the production of pro-inflammatory mediator.

As an inflammation-related transcription factor, STAT1 protein plays an important role in mediating inflammatory responses by regulating a variety of cellular processes and modulating target gene expression, cell proliferation, survival, apoptosis, and differentiation [39, 40]. The noxious effect of IFN-γ is mediated by the activation of STAT1, which induces inflammatory cell infiltration by the expression of leukocyte-attracting chemokines [41]. On the other hand, STAT1 was most strongly activated along Bowman’s capsule epithelial cells and in some cells along the periphery of glomeruli, accelerating the progression of glomerulosclerosis, thus having a deleterious effect on kidney physiology and function [40]. The activation and nuclear localization of STAT1 transcription factors is primarily dominated by tyrosine phosphorylation, dimerization and translocation to the nucleus [42]. Our results have shown that KJY exerted a dramatic role in inhibiting expression of phosphorylated (activated) STAT1 (p-STAT1) induced by adenine *in vivo* and LPS *in vitro*. It is in line with the previous finding that blockade of STAT1 activity reduces macrophage infiltration and improves renal function in mice with lupus nephritis [43].

In conclusion, the combination of K, J, and Y had the strongest effect on recovering the levels of renal biochemical indicators and the expression of the ECM proteins in CRF rat models. In most cases, the combination therapy of any 2 of K, J, and Y achieved greater improvements than single drug treatments alone. Hence, the current study provided the first evidence that a prescription with K, J, and Y has compelling rationality. Moreover, our study indicated that KJY exerted considerable therapeutic effects on inhibiting STAT1 activation to reduced the secretion of inflammatory cytokines and improve the renal function of rats with adenine-induced chronic renal failure.

## MATERIALS AND METHODS

### Materials

#### Drugs

Chitosan was purchased from Bozhihui Biological Technology Co., Ltd. (Qingdao, Shandong, China), which has a purity of 90%. Gynostemma and Motherwort extracts, both brought from Sinuote Biological Technology Co., Ltd. (Xi’an, Shanxi, China), containing 80% of Gypenosides and Leonurine, respectively. Adenine was purchased from Aladdin Industrial Corporation (Los Angeles, Southern California, USA).

#### Testing kits

The following assay kits (Jiancheng Ltd., Nanjing, Jiangsu, China) were used: BUN detection kit (urease method, lot# 20150514), CRE detection kit (sarcosine oxidase method, lot# 20150330), and detection kits for uric acid(UA) (lot# 20150428), hemoglobin (HB) (lot# 20150504), albumin (ALB) (lot# 20150504), malondialdehyde (MDA) (lot# 20150512),γ-glutamyl transferase (γ-GT), glutathione (GSH) (lot# 20150514), glutathione peroxidase (GSH-PX) (lot# 20150511), and superoxide dismutase (SOD) (lot# 20150320). Mouse Interleukin-1 Beta (IL-1β), Tumor necrosis factor-α (TNF-α), Interferon-gamma (IFN-γ), chemokine (C-X-C motif) ligand 10 (CXCL10) and COX-2 ELISA kits were purchased from Nanjing Aoqing Co., Ltd (Nanjing, Jiangsu, China).

#### Antibodies

Monoclonal anti-collagen I, anti-TIMP-1 and anti-matrix metalloprotease-1(MMP-1) were obtained from Signalway (Pirland, Texas, USA), and monoclonal anti-plasminogen activator inhibitor 1(PAI-1) was from KeyGEN Ltd., (Nanjing, Jiangsu, China). Monoclonal anti-β-actin, anti-p-STAT1, anti-COX-2 and anti-iNOS were purchased from Cell Signaling Technology (Danfoss, Massachusetts, USA).

#### Cell line and reagents

Mouse RAW264.7 macrophages were purchased from Wuhan Procell Co., Ltd (Wuhan, Hubei, China). LPS was purchased from Sigma Aldrich (St. Louis, Missouri, USA).

### *In vivo* study

#### Animals

Male Sprague-Dawley (SD) rats (250 to 300 g) were provided by the experimental animal center of ZheJiang Province. The animals were housed in standard polypropylene cages and given adequate clean water and food. The room temperature was controlled at 21–23°C, with a relative humidity of 50–60%. Procedures involving animals and their care were in accordance with China state regulations on animal experimentation and were approved by the Animal Experimental Ethical Center of Southeast University (protocol number 20120023).

#### Experimental protocol

The experimental CRF model was established by oral administration with 250 mg/kg bodyweight (b.w.) adenine suspension (Aladdin^®^, Los Angeles, CA, USA; 50 mg/ml, suspended in 0.5% CMC-Na at a dosing volume of 0.50 ml/100 g) for 4 consecutive weeks. Briefly, adenine was given once a day for the first 2 weeks, then the same dose was given once every other day for the next 2 weeks. At the same time, rats in the control group received 0.5% CMC-Na solution (0.50 ml/100 g b.w.) orally. Serum levels of BUN and CRE were measured at the end of treatment to confirm the successful construction of the model.

The BUN value is regarded as the main criteria for the successful establishment of the model of chronic renal failure. Rats in the model group that achieved BUN values of 20–54 were included in the study. Model and control rats were divided into 9 groups (*n* = 8 per group). As shown in Table 2, apart from the control (Ctrl) and model (Mod) groups, the remaining groups received different combinations of chitosan (represented by K), gynostemma (represented by J), and motherwort extract (represented by Y). The purity of K was 90%, and the J and Y extracts contained 80% gypenosides and leonurine, respectively. Different combinations of the three drugs were mixed homogeneously at various proportions (shown in Table 2). The dose of each drug in each group was the same. The Ctrl and Mod groups were treated orally with 0.5% CMC-Na solution (used to dissolve the drugs) at a volume of 0.5 ml/dose. All treatments were given twice daily (in the morning and afternoon at 8-hour intervals) for 5 weeks. Bodyweight and food intake were monitored once per week during the experimental period.

**Table 2:** The drug administration doses in each group

Group	K (g)	J (g)	Y (g)	Total dose (g/kg b.w./day)
Ctrl	0	0	0	0
Mod	0	0	0	0
K	0.325	0	0	0.325
J	0	0.1	0	0.100
Y	0	0	0.205	0.205
KJ	0.325	0.1	0	0.425
KY	0.325	0	0.205	0.530
JY	0	0.1	0.205	0.305
KJY	0.325	0.1	0.205	0.630

After two-week of drug administration, blood was collected by tail bleeding. Serum levels of BUN and CRE in each group were examined. At the end of the experiment (5 weeks after administration), rats were anesthetized using urethane (1.0 g/kg b.w.), and blood was taken via the aorta. The blood samples were centrifuged at 4000 rpm for 15 min, and serum was separated and stored at −80°C until they were used to be examined for the serum biochemical indicators. Kidney tissues were removed carefully; one was stored at −80°C to measure biochemical indicators and perform Western blotting. The other one was fixed in 4% formaldehyde solution for HE staining and immunohistochemistry.

#### Biochemical analysis

The levels of biochemical indicators including serum BUN, CRE, UA, HB, ALB and kidney malondialdehyde MDA, γ-GT, GSH, GSH-PX, and SOD were examined using commercial kits (Jiancheng Ltd., Nanjing, Jiangsu, China). The enzyme reaction products reflecting the concentrations of the biochemical indicators were read using a full-wave microplate reader.

#### Histological observations

The second kidney excised from each animal was fixed in 4% formaldehyde solution for at least 48 h, dehydrated in ethanol, cleared with xylene, and embedded in paraffin. Then, a Leica microtome (Germany) was used to cut 3-μm sections, which were stained with hematoxylin and eosin (H&E). Finally, the slides were evaluated by a histopathologist using the method reported by Marin et al [20]. Three indices were used to assess the degree of renal injury: the amount of pathological damage, renal tubular lesions, and interstitial lesions.

#### Western blotting

Tissue samples were homogenized with an electric homogenizer at 1000 rpm in lysis solution (including PMSF). They were then centrifuged at 12000 rpm for 5 min at 4°C and the supernatants were aliquot as crude protein samples. BCA reagent (Thermo Fisher Scientific, USA) was used to measure protein concentrations. Proteins samples were separated using SDS gel electrophoresis and transferred to nitrocellulose membranes. Non-specific binding was blocked by incubation in 5% bovine serum albumin (BSA) overnight at 4°C. Then, the membranes were incubated with primary antibodies (monoclonal anti-collagen I, anti-TIMP-1, anti-MMP-1, anti-PAI-1, anti-COX-2, anti-iNOS, anti-p-STAT1 and anti-β-actin) overnight at 4°C. After rinsing, the membranes were exposed to goat anti-mouse and anti-rabbit IgG horseradish peroxidase (HRP)-conjugated secondary antibodies for 2 h at room temperature. Finally, the signals were detected using a chemiluminescent method (ECL, Tanon-5200).

### *In vitro* study

#### MTT assay

RAW 264.7 cells (1 x 10^5^ / well) were seeded on 96-well plates and incubated overnight with 5% CO2, at 37°C, until the cell density was 80% to 90%. The medium was discarded followed by adding culture medium containing the drugs for 12 h. After that, the supernatant was collected and used for ELISA assay. Then, fresh medium and 20 μL of MTT solution were added to each well and incubated at 37° C for 4 h. After discarding the supernatant, 150 μL of DMSO was added to each well to dissolve the crystal violet. Light absorbance (OD) was measured by enzyme immunoassay at 570 nm (490 nm as a reference). Each group of experimental samples was put into three wells. Grouped as follows: (1) Cell + culture medium; (2) Cell+LPS (1 μg/mL); (3) Cell+LPS (1 μg/mL)+ KJY (31 μg/mL); (4) Cell+LPS (1 μg/mL)+ KJY (62 μg/mL); (5) Cell+LPS (1 μg/mL)+ KJY (93 μg/mL); (6) Cell+LPS (1 μg/mL)+ KJY (186 μg/mL); (7) Cell+LPS (1 μg/mL)+ KJY (248 μg/mL).

#### ELISA

ELISA kits were applied to detect secretion of IL-1β, TNF-α, IFN-γ, CXCL10 and COX-2 levels. Firstly, 50 μL of the standard or 5 times dilution of the sample was added to each well and incubated for 30 min at 37 °C. After discarding the liquid, wells were washed 5 times with washing solution and dried. Then, 50 μL of enzyme-labeled reagent was added to each well and incubated at 37°C for 30 min. After wash, 50 μL of reagent A was added to each well then 50 μL of reagent B was added. Wells were incubated for 15 min at 37°C under dark. Then, 50 μL of stop solution was added to terminate the reaction. The light absorbance (OD) of each well was measured at 450 nm immediately. The actual concentration of the sample is calculated from the standard curve.

#### RNA isolation and real-time PCR

Total RNA was prepared from cultured cells using the Trizol reagent (TaKaRa, Japan) according to the manufacturer’s instructions. Single-stranded cDNAs were synthesized and real time PCR was performed, as described previously (11). Reactions were run on a RT-PCR system (ABI Prism 7500; Applied Biosystems, USA). Gene expression was detected with SYBR Green (Ta-KaRa, Japan) and GAPDH was chosen as a housekeeping gene for normalization of relative gene expression. The PCR products were sequenced to validate the identity of the amplicons. The mRNA expression of COX-2 and iNOS in RAW264.7 macrophages treated with different drugs, was normalized to its expression level in control molecules and calculated from the 2^-ΔΔCt^ method.

#### Western blotting

Protein expression of COX-2、iNOS and p-STAT1 in RAW264.7 macrophages was analyzed by Western blotting. The method was as described above in *in vivo* study.

### Statistical analysis

The results are reported as mean ± SDs (standard deviations) and were analyzed using one-way ANOVA. LSD’s multiple comparison tests were used for comparisons among groups after the overall analysis of variance. Differences were considered significant at *P<*0.05 and extremely significant at *P<*0.01. Furthermore, a 2 × 2 × 2 mixed-factor ANOVA model was used to assess the interactions among K, J, and Y for the treatment of CRF.
